# Biomechanical Behavior of Narrow Dental Implants Made with Aluminum- and Vanadium-Free Alloys: A Finite Element Analysis

**DOI:** 10.3390/ma15248903

**Published:** 2022-12-13

**Authors:** José Manuel Zapata, Eduardo Leal, Renato Hunter, Raphael Freitas de Souza, Eduardo Borie

**Affiliations:** 1 Master in Dental Sciences Program, Universidad de La Frontera, Temuco PC 4811230, Chile; 2 Mechanical Engineering Department, Universidad de La Frontera, Temuco PC 4811230, Chile; 3 Faculty of Dental Medicine and Oral Health Sciences, McGill University, Montreal, QC H3A 0G4, Canada; 4 CICO Research Centre, Integral Dentistry Department, Dental School, Universidad de La Frontera, Temuco PC 4811230, Chile

**Keywords:** biomechanics, bone–implant interface, dental alloys, single tooth dental implant

## Abstract

Titanium (Ti) alloys used for narrow dental implants usually contain aluminum (Al) and vanadium (V) for improved resistance. However, those elements are linked to possible cytotoxic effects. Thus, this study evaluated the biomechanical behavior of narrow dental implants made with Al- and V-free Ti alloys by the finite element method. A virtual model of a partially edentulous maxilla received single implants (diameter: 2.7 and 2.9 mm; length: 10 mm) at the upper lateral incisor area, with respective abutments and porcelain-fused-to-metal crowns. Simulations were performed for each implant diameter and the following eight alloys (and elastic moduli): (1) Ti–6Al–4V (control; 110 GPa), (2) Ti–35Nb–5Sn–6Mo–3Zr (85 GPa), (3) Ti–13Nb–13Zr (77 GPa), (4) Ti–15Zr (113 GPa), (5) Ti–8Fe–5Ta (120 GPa), (6) Ti–26.88Fe–4Ta (175 GPa), (7) TNTZ–2Fe–0.4O (107 GPa), and (8) TNTZ–2Fe–0.7O (109 GPa). The implants received a labially directed total static load of 100 N at a 45° angle relative to their long axis. Parameters for analysis included the maximum and minimum principal stresses for bone, and von Mises equivalent stress for implants and abutments. Ti–26.88Fe–4Ta reaches the lowest maximum (57 MPa) and minimum (125 MPa) principal stress values, whereas Ti–35Nb–5Sn–6Mo–3Zr (183 MPa) and Ti–13Nb–13Zr (191 MPa) models result in the highest principal stresses (the 2.7 mm model surpasses the threshold for bone overload). Implant diameters affect von Mises stresses more than the constituent alloys. It can be concluded that the narrow implants made of the Ti–26.88Fe–4Ta alloy have the most favorable biomechanical behavior, mostly by mitigating stress on peri-implant bone.

## 1. Introduction

Contemporary dental implants systems have reached excellent levels of predictability, as evidenced by their high survival rates [1,2,3]. This is true not only for standard diameter implants, but also for narrow fixtures. Potential benefits of narrow implants include the treatment of edentulous spaces with reduced mesiodistal or buccolingual dimensions without previous bone augmentation or orthodontic movement. Despite the several existing classification systems, implants of less than 3.5 mm of diameter are considered narrow [4,5]. Implants of less than 3.0 mm can be classified as extra-narrow, and are often used in areas of low occlusal loads, e.g., upper lateral incisors and lower incisors [4,6,7].

Narrow implants pose a special biomechanical challenge and, thus, need to be fabricated with highly resistant alloys to compensate for the reduction in diameter. Whereas grade 4 Ti alloys combine excellent biocompatibility and acceptable mechanical properties when used in standard implants [8,9], narrow implants need stronger alloys to resist fracture [10,11,12]. Most manufacturers choose grade 5 Ti or Ti–6Al–4V alloys for narrow fixtures, due to their lower cost and ease of machining [13].

Even if Ti–6Al–4V presents good biomechanical properties, its use brings some controversies. Issues with this alloy include a possible link between aluminum alloys and neurodegenerative diseases [14,15,16], allergies, neurological disorders [17,18], and the potential cytotoxicity of vanadium ions [13]. It is important to highlight that even non-toxic concentrations of these ions could be enough to produce biological changes [19].

Even if zirconia implants have been purported as substitutes for Ti–6Al–4V fixtures [20], they have major limitations due to the reduced diameters. For instance, 3.0 mm wide, one-piece zirconia implants show important fatigue failure rates [21]. Extra-narrow implants still demand the flexibility of Ti in alloys with adequate resistance and biocompatibility. In this sense, less toxic elements such as zirconium, niobium, tantalum, molybdenum, and iron can be added to Ti alloys to increase their strength [22,23]. This opens a potential improvement for narrow dental implants, by combining optimal resistance and good biocompatibility. To the best of our knowledge, the biomechanical testing of narrow dental implants made with those elements is unprecedented. Thus, this study evaluated the biomechanical behavior of narrow dental implants with Al- and V-free alloys by the finite element method, as a first step to support their use for implant manufacturing. The null hypothesis is that the tested Al- and V-free alloys do not result in an improvement in stress distribution in narrow implants, compared to Ti–6Al–4V.

## 2. Materials and Methods

This in silico study tested a model composed of a partially edentulous maxilla restored by narrow implants and respective single crowns.

A DICOM file depicting a case with a partially edentulous maxillary arch (missing teeth: lateral incisors) was obtained from a tomography database (CTI, Campinas, SP, Brazil) and transformed into the .step format using the Invesalius v. 3.0 (CTI, Campinas, SP, Brazil). The three-dimensional .step model was exported to Rhinoceros SR8 v.6.0 software (McNeel North America, Seattle, WA, USA), refined, and limited to the anterior region of the edentulous maxilla. The model was designed to present a cortical bone thickness of 1 mm surrounding trabecular bone, corresponding to Lekholm and Zarb type III bone [24].

Tridimensional images of implants and abutments were obtained by scanning them directly. Conometric connection implants (Helix GM, Neodent, Curitiba, Brazil) and their respective abutments (Pilar GM, Neodent, Curitiba, Brazil) were cut with an electro-erosion machine (FW 1U, AgieCharmilles, GF Machining Solutions, Beijing, China), and each geometry was obtained through a 3D scanner (Capture Mini, 3D Systems, Rock Hill, SC, USA), being transformed into the 0.3 dm format. The surface of each component was opacified with penetrant ink to capture their details (Spotcheck, SKL-SP2 Penetrant, Magnaflux, IL, USA). Once the geometries of the implants and abutments were obtained, they were scaled, obtaining implants with a diameter of 2.7 and 2.9 mm, both 10 mm in length.

In turn, the models received the implants at the position of the missing upper lateral incisor, in compliance with recommendations for narrow implants. All models had the implants placed at the same angulation and at bone level. Subsequently, each implant received a solid one-piece abutment and cement-retained porcelain-fused-to-metal single crown (Co–Cr framework covered by feldespatic ceramic). The latter was shaped according to the expected natural shape of an upper lateral incisor.

The simulations were divided into groups according to the two diameters and eight alloys to be analyzed as implant constituents, as well as abutments: (1) Titanium grade V (Ti–6Al–4V; control); (2) titanium–niobium–tin–molybdenum–zirconium (Ti–35Nb–5Sn–6Mo–3Zr); (3) titanium–niobium–zirconium (Ti–13Nb–13Zr); (4) titanium–zirconium (Ti–15Zr); (5) titanium–iron–tantalum (Ti–8Fe–5Ta); (6) titanium–iron–tantalum with a higher concentration of iron (Ti–26.88Fe–4Ta); (7) titanium–niobium–tantalum–zirconium–iron–oxygen (TNTZ–2Fe–0.4O); (8) titanium–niobium–tantalum–zirconium–iron–oxygen with higher oxygen concentration (TNTZ–2Fe–0.7O). The modulus of elasticity and Poisson’s ratio of each material were obtained from the literature (Table 1).

**Figure 1:**
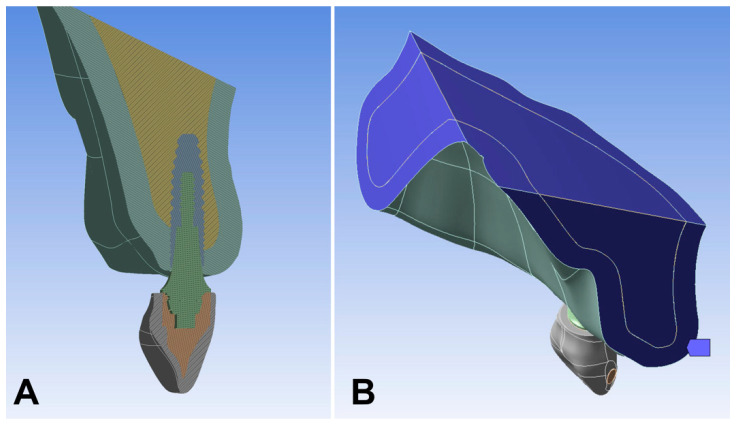
(**A**) Different layers of the model; (**B**) boundary conditions.

Finite element model simulation was performed by exporting the models to Ansys Workbench version 19.0 software (Ansys Inc., Canonsburg, PA, USA). All structures were considered to be isotropic, homogeneous, and linearly elastic. To simplify data analysis, only half of the model was analyzed by the software, divided by the mid-sagittal plane, i.e., the section plane passed symmetrically through the mesial contact of the upper central incisors and median palatine suture. Subsequently, the mesh was refined mainly at the bone–implant interface and on the prosthetic components. The gingiva and the cement were not considered in the models due to their null influence on the analysis [36]. All abutments were fixed to the implant based on a perfect adaptation and with a perfect union (Figure 1A), while boundary conditions are shown in Figure 1B. Subsequently, a total static oblique load of 100 N was applied to the cingulum area of the palatal surface of the incisor, at an angle of 45° with respect to the long axis of the implant in the buccal direction [37]. Trabecular–cortical bone, implant–bone, implant–abutment, abutment–framework, and framework–restoration ceramic interfaces were considered as bonded contacts. Furthermore, the implant was considered to be completely osseointegrated.

The total deformation of each model was initially evaluated and convergence tests were performed to check analytical accuracy. For quantitative analysis, the implants and abutments were assessed with von Mises equivalent stress (VMES), while the bone was analyzed by maximum and minimum principal stresses. The graphic display of color maps was used to compare the models qualitatively. All graphic scales were standardized according to each model. Thresholds of 100 MPa for tensile or maximum principal stresses and 170 MPa for compressive or minimum principal stresses were considered as evidence of bone overload [38].

## 3. Results

The stress values observed in the peri-implant bone, implant, and abutment for each alloy in the diameters tested are summarized in Table 2 and Table 3.

Models of Ti–15Zr, TNTZ–2Fe–0.4O, and TNTZ–2Fe–0.7O alloys show maximum and minimum principal stresses values comparable to Ti–6Al–4V (control), while Ti–8Fe–5Ta has lower principal stress values.

**Figure 2:**
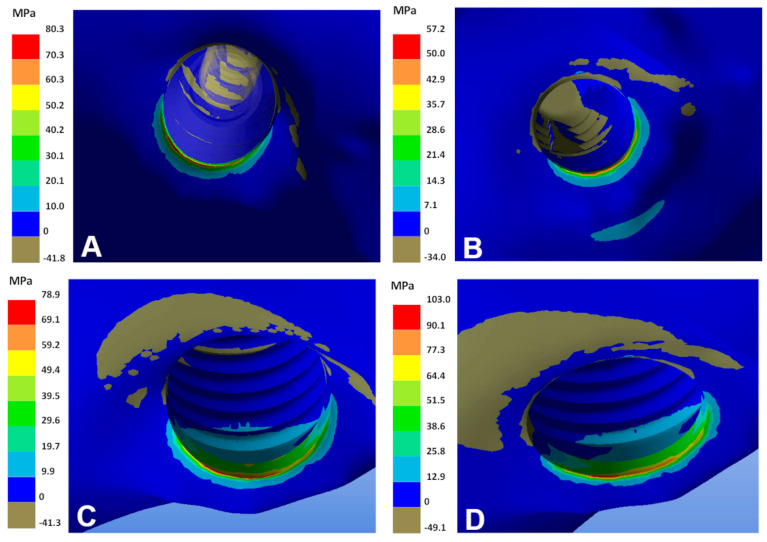
Maximum principal stresses in the bone of the different models. (**A**) Ti–6Al–4V; (**B**) Ti–26.88Fe–4Ta; (**C**) Ti–15Zr; (**D**) Ti–13Nb–13Zr.

**Figure 3:**
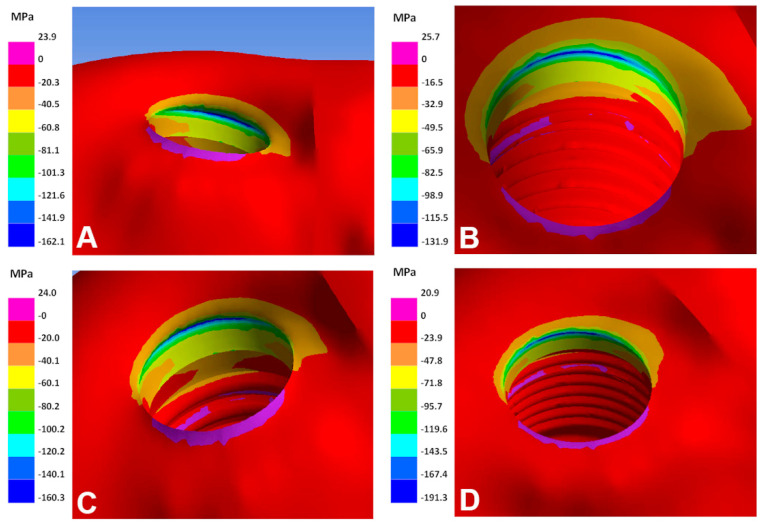
Minimum principal stresses in the bone of different implant alloys. (**A**) Ti–6Al–4V; (**B**) Ti–26.88Fe–4Ta; (**C**) Ti–15Zr; (**D**) Ti–13Nb–13Zr.

The lowest maximum and minimum principal stress values are identified with the two models of the Ti–26.88Fe–4Ta alloy. The highest principal stresses at the peri-implant region are observed with the models of Ti–35Nb–5Sn–6Mo–3Zr and Ti–13Nb–13Zr alloys (Figure 2 and Figure 3). The combination between these two alloys (Ti–35Nb–5Sn–6Mo–3Zr and Ti–13Nb–13Zr) and the smallest diameter (2.7 mm implants) results in bone overload, i.e., stress beyond 170 MPa.

**Figure 4:**
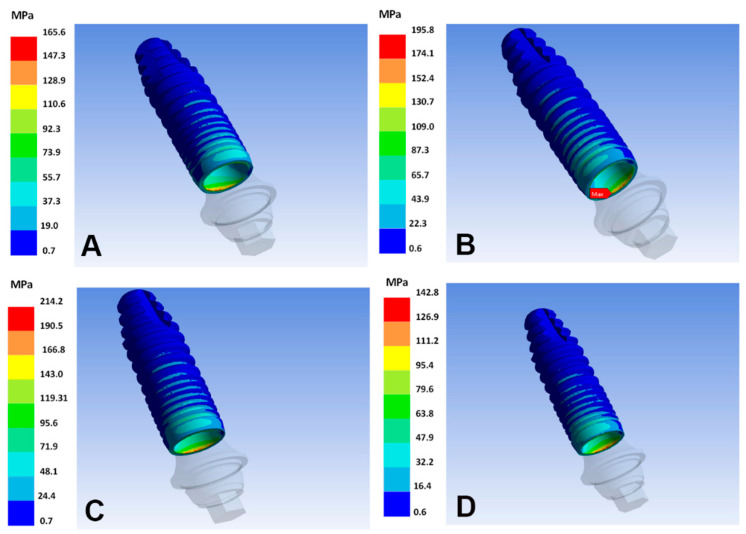
von Mises equivalent stresses in the implant surface of the different alloys. (**A**) T–6Al–4V; (**B**) Ti–26.88Fe–4Ta; (**C**) Ti–15Zr; (**D**) Ti–13Nb–13Zr.

**Figure 5:**
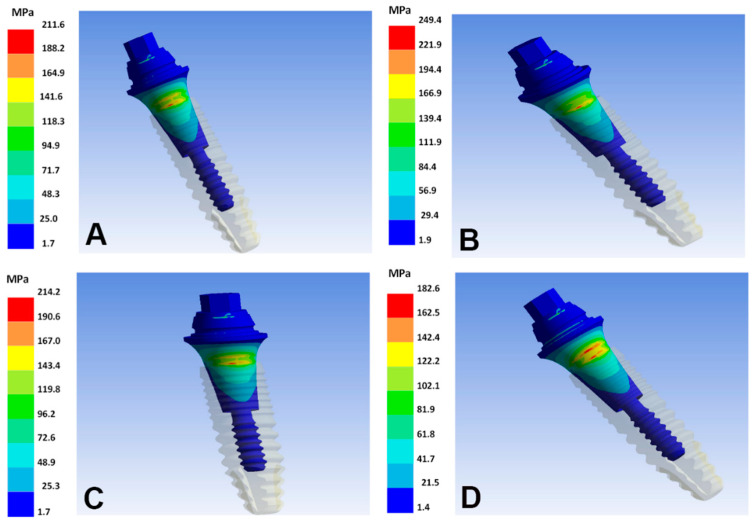
von Mises equivalent stresses in the abutment surface of the different alloys. (**A**) Ti–6Al–4V; (**B**) Ti–26.88Fe–4Ta; (**C**) Ti–15Zr; (**D**) Ti–13Nb–13Zr.

Ti–35Nb–5Sn–6Mo–3Zr and Ti–13Nb–13Zr reach the lowest VMES in the implants, regardless of the diameter. Minimum VMES in abutments is observed in the Ti–13Nb–13Zr and Ti–15Zr alloys for the implants of 2.7 mm and 2.9 mm, respectively. Regarding the implants, the highest VMES is identified in the following two combinations: Ti–26.88Fe–4Ta with 2.7 mm diameter, and Ti–15Zr with 2.9 mm diameter (Figure 4). For the abutments, the highest VMES is observed with the Ti–26.88Fe–4Ta alloy, regardless of the diameter (Figure 5).

In the ductile materials (i.e., implant and abutment), the highest VMES of all the models is identified in the joint of the implant–abutment (Figure 5). Also, none of the analyzed VMES values achieve the ultimate strength values (fracture) in the abutment or the implant.

## 4. Discussion

This study was a first step to identify alternative alloys for narrow dental implants, without aluminum and vanadium. The null hypothesis was rejected, given that some of the tested Al- and V-free alloys improve stress distribution in narrow dental implants.

The present finite element analysis demonstrates that some alloys can be used in lieu of the traditional Ti–6Al–4V and, thus, have acceptable biomechanical behavior in narrow implants, at least for specific indications (e.g., single replacement for lateral and lower incisors) [39,40]. With the current development of 3D additive manufacturing of biomedical implants [41,42,43], the manufacture of narrow dental implants with high-strength Al- and V-free titanium alloys may become commonplace. However, this study did not evaluate the time, facility, and feasibility of manufacturing narrow dental implants with each tested alloy.

The alloys made of Ti–15Zr, Ti–8Fe–Ta, Ti–26.88Fe–4Ta, TNTZ–2Fe–0.4O, and TNTZ–2Fe–0.7O show values within physiological ranges, in compliance with the bone overload risk values of tensile (100 MPa [38]) and compressive stresses (170 MPa [38]). However, the minimum stresses for the Ti–35Nb–5Sn–6Mo–3Zr and Ti–13Nb–13Zr alloys surpass the physiological ranges. This overload could result in bone resorption and imbalance in the apposition–resorption process, as bone tends to gradually change its morphology in an attempt to adapt to the load [44,45]. The ultimate consequence would be a failure in osseointegration, with implant loss. Therefore, we do not recommend further analysis or tests with these latter alloys for extra-narrow implants (diameter: 2.9 mm or less), due to the increased risk of bone overload. No alloy reaches yield/ultimate stress during the analysis, which indicates good resistance of the implant per se, regardless of the alloy.

The alloys of Ti–15Zr, TNTZ–2Fe–0.4O, and TNTZ–2Fe–0.7O show similar maximum and minimum principal stresses values as Ti–6Al–4V (control) in both implant diameters. It means that these alloys can be as alternatives to replace Ti–6Al–4V, due to their similar biomechanics’ behavior in narrow dental implants.

Also, this research shows that in the alloys with a higher elastic modulus, higher magnitudes of von Mises stress are observed in the abutments and implants, but a better distribution to the surrounding bone, which concurs with Datte et al. [20]. This is relevant, since the most resistant materials finally absorb the stresses [46], reducing the risk of bone overload.

Our study confirms that adding Fe to titanium alloys is positive for resistance, even though the diameter is reduced. As previously described, Fe increases the yield strength and resistance in vitro [13]. However, despite the fact that tensile and compressive stresses are higher for the Ti–Zr alloy than for Ti–26.88Fe–4Ta, it could be within the physiological limits in the 2.9 and 2.7 mm implants. Interestingly, the addition of niobium and zirconium is linked to a lower elastic modulus when compared with the widely used Ti–6Al–4V alloys. This would make those alloys a poorer option for extra-narrow implants.

The analysis of this study was static, however, whereas a dynamic analysis may display values 10–20% higher [47]. In this sense, the most indicated alloy for 2.7 and 2.9 mm is Ti–26.88Fe–4Ta, due to a security margin from the physiological ranges (100 MPa/170 MPa) during dynamic behavior. Also, it is important that implants and abutments are made of the same alloy, and do not have differences, to promote better stress distribution [46].

A limitation of this study was the focus on a single implant length. However, implant diameter is a more effective factor than implant length to improve the stress distribution in the surrounding bone [38,48]. It is logical to think that narrow implants shorter than 10 mm may increase the risk of bone overload and should not be used. Other potential limitation included the use of porcelain-fused-to-metal crowns in all models. However, it is known that the crown material has negligible influence in the stress distribution of a whole system [20].

It is important to highlight that this study tackles a specific aspect (i.e., expected stress distribution), but it has relevant results as the first step for further analysis of the proposed alloys to be used in narrow implants. Considering the limitations of this in silico study, further studies are necessary to determine the most suitable alloy to be used in narrow implants, considering the feasibility in its manufacture, as well as their in vitro biomechanical behavior and its biological safety. The long-term success of dental implants smaller than 3 mm is not documented in the literature [4,49], even if they are appealing for use, such as in lateral incisors.

## 5. Conclusions

Considering the distribution of the stresses to the bone surrounding narrow dental implants, Ti–26.88Fe–4Ta is the most favorable alloy, whereas the Ti–35Nb–5Sn–6Mo–3Zr and Ti–13Nb–13Zr models surpass the bone overload threshold. The Ti–26.88Fe–4Ta alloy has promising use for the rehabilitation of small diameter teeth and should be further tested for dental applications.

## Figures and Tables

**Table 1:** Mechanical properties of the materials.

Material	Young’s Modulus (GPa)	Poisson Ratio	Ultimate Strength (MPa)
Cortical bone	13.7 [25]	0.3 [25]	100–130 [26]
Trabecular bone	0.5 [27]	0.3 [27]	5 [28]
Ti–6Al–4V	110 [29]	0.35 [29]	1000 [8]
Ti–35Nb–5Sn–6Mo–3Zr	85 [8]	0.34 [8]	770 [8]
Ti–13Nb–13Zr	77 [30]	0.34 [30]	1270 [31]
Ti–15Zr	113 [8]	0.34 [8]	N/A [8]
Ti–8Fe–5Ta	120 [8]	0.34 [8]	1045 [8]
Ti–26.88Fe–4Ta	175 [8]	0.34 [8]	2531 [8]
TNTZ–Fe–0.7O	109 [32]	0.35 [32]	N/A [32]
TNTZ–Fe–0.4O	107 [32]	0.35 [32]	1130 [32]
Co–Cr	205 [33]	0.31 [33]	655 [34]
Feldespatic ceramic	68.9 [35]	0.28 [35]	N/A [29]

**Table 2:** Principal stresses and VMES values for alloy simulated in the implant of 2.7 mm in diameter.

Implant Alloy	Maximum Principal, Bone (MPa)	Minimum Principal, Bone (MPa)	VMES, Implants (MPa)	VMES, Abutments (MPa)
**Ti–6Al–4V**	80	−162	165	211
**Ti–35Nb–5Sn–6Mo–3Zr**	96	−183 *	149	190
T**i–13Nb–13Zr**	103	−191 *	142	182
**Ti–15Zr**	79	−160	168	214
**Ti–8Fe–5Ta**	75	−155	171	219
**Ti–26.88Fe–4Ta**	58	−131	195	249
**TNTZ–2Fe–0.4O**	82	−164	163	209
**TNTZ–2Fe–0.7O**	81	−163	165	210

* Surpasses the threshold for bone overload (±170 MPa).

**Table 3:** Principal stresses and VMES values for alloy simulated in the implant of 2.9 mm in diameter.

Implant Alloy	Maximum Principal, Bone (MPa)	Minimum Principal, Bone (MPa)	VMES, Implants (MPa)	VMES, Abutments (MPa)
**Ti–6Al–4V**	81	−149	143	176
**Ti–35Nb–5Sn–6Mo–3Zr**	93	−164	117	183
**Ti–13Nb–13Zr**	101	−170 *	128	187
**Ti–15Zr**	79	−147	178	145
**Ti–8Fe–5Ta**	75	−144	148	182
**Ti–26.88Fe–4Ta**	57	−125	161	203
**TNTZ–2Fe–0.4O**	80	−150	122	180
**TNTZ–2Fe–0.7O**	79	−164	123	181

* Achieve the threshold for bone overload (±170 MPa).
